# Assessment of polytrauma patient management in India

**DOI:** 10.6026/973206300212618

**Published:** 2025-08-31

**Authors:** Piyush Pratap Singh Sikarwar, Jay Kumar Kolewar, Devendra Sharma, Sandeep Kumar Dwivedi, Akanksha Sikarwar

**Affiliations:** 1 Department of Emergency Medicine, Gajra Raja Medical College, Gwalior, Madhya Pradesh, India; 2 Department of Anaesthesiology, Gajra Raja Medical College, Gwalior, Madhya Pradesh, India; 3 Department of Orthopedic and Trauma, Gajra Raja Medical College, Gwalior, Madhya Pradesh, India; 4 Department of Periodontology, Maharana Pratap College of Dentistry and Research, Centre, Gwalior, Madhya Pradesh, India

**Keywords:** Polytrauma, ATLS, emergency care, imaging delay, transfusion ratio, trauma team, injury severity score

## Abstract

ATLS adherence in 250 polytrauma patients in India is of interest. While airway control and cervical spine immobilization were timely, delays in CT imaging and suboptimal transfusion ratios (PRBC: FFP = 1.5:1) were noted. Patients with ISS > 25 had significantly longer imaging times and higher mortality (21% vs. 9%). Overall, in-hospital mortality was 14%. Dedicated trauma teams and protocol optimization are recommended for improved outcomes.

## Background:

Polytrauma, defined as multiple simultaneous injuries involving at least two body regions with potential life-threatening implications, remains a leading cause of morbidity and mortality worldwide, particularly among young adults [1]. The initial minutes of care-often referred to as the "Golden Hour"-are critical for preventing the lethal triad of coagulopathy, hypothermia and acidosis [2]. Standardized approaches such as the Advanced Trauma Life Support (ATLS) system were developed to provide a structured algorithm for airway, breathing, circulation, disability, and exposure (ABCDE) assessment [3]. Despite widespread adoption, studies reveal variability in adherence to ATLS guidelines, with significant implications for patient outcomes [4, 5]. Recent investigations have highlighted that delays in airway management, imaging and blood product administration contribute to elevated mortality rates in high-severity cases [6, 7]. Moreover, the lack of a dedicated multidisciplinary trauma team often results in fragmented care pathways and prolonged emergency department (ED) and length of stay (LOS) [8]. In low- and middle-income settings, resource constraints further exacerbate delays in diagnostics and definitive care [9]. Therefore, it is of interest to assess adherence to ATLS protocols in the ED management of polytrauma patients and to determine factors associated with delays and increased mortality.

## Materials and Methods:

## Study design:

A prospective observational study was conducted in the ED of a tertiary care hospital between January and June 2025.

## Sample size and selection:

Consecutive adult polytrauma patients (age ≥ 18 years) presenting within 6 hours of injury were enrolled. Exclusion criteria included isolated single-system injuries and patients declared dead on arrival. A sample size of 250 was determined to provide 80% power to detect a 10% difference in mortality between high- and low-severity groups.

## Equipment and materials:

Standard ATLS equipment was utilized, including advanced airway kits, portable ultrasound with Focused Assessment with Sonography for Trauma (FAST), and a 64-slice CT scanner. A massive transfusion protocol (MTP) kit containing predefined ratios of packed red blood cells (PRBCs), fresh frozen plasma (FFP), and platelets was available.

## Experimental procedures:

Upon arrival, patients underwent ABCDE assessment. Time to airway control (endotracheal intubation or supraglottic device), cervical spine immobilization, initial FAST, and transfer to CT were recorded. Decisions on blood product administration followed the institutional MTP: PRBC: FFP: platelet ratio target of 1:1:1. Post-ED disposition (ICU admission or operating room) and total ED LOS were documented.

## Statistical methods:

Data distribution was assessed via the Shapiro-Wilk test. Continuous variables are expressed as mean ± SD and compared using Student's t-test. Categorical variables are presented as percentages and compared using chi-square or Fisher's exact test. A p-value < 0.05 was considered statistically significant. Statistical analyses were performed using SPSS v27.0.

## Results:

A total of 250 patients were enrolled (Table 1 - see PDF). The mean age was 34 ± 12 years, and 170 (68%) were male. Mechanisms of injury included road traffic accidents (56%), falls from height (32%), and other causes (12%). Mean ISS was 24 ± 8, with 105 (42%) patients having ISS > 25. Time to airway control averaged 8 ± 3 minutes; 225 (90%) patients received cervical spine immobilization. Initial FAST was performed in 210 (84%) patients within 6 ± 2 minutes of arrival. Median door-to-CT time was 22 ± 6 minutes (Table 2 - see PDF). One hundred and five patients (42%) received blood products. The mean PRBC: FFP ratio was 1.5 ± 0.3, and platelets were administered in 38 (36%) of transfused patients. Mean transfusion volume in the first 24 hours was 4.2 ± 2.1 units PRBC and 3.1 ± 1.5 units FFP (Table 3 - see PDF). Overall, ED LOS was 3.8 ± 1.2 hours. One hundred and fifty (60%) patients were admitted to the ICU, and 55 (22%) proceeded directly to the operating room. Seventy (28%) were managed in the HDU or ward after initial stabilization. In-hospital mortality was 14% (n = 35). Patients with ISS > 25 experienced longer median door-to-CT times (25 ± 7 vs. 20 ± 5 minutes; p = 0.002) and higher mortality (21% vs. 9%; p = 0.01) compared to those with ISS ≤ 25 (Table 3 - see PDF). There was no significant difference in time to airway control between groups (8 ± 3 vs. 7 ± 2 minutes; p = 0.08) (Table 4 - see PDF).

## Discussion:

This study highlights both the strengths and critical gaps in the adherence to Advanced Trauma Life Support (ATLS)-based management within a high-volume tertiary emergency department (ED). While early interventions such as airway management and cervical spine immobilization were efficiently implemented in the majority of cases-demonstrating strong compliance with primary survey protocols-downstream elements of trauma care, including imaging and transfusion strategies, revealed opportunities for substantial improvement, particularly among patients with high Injury Severity Scores (ISS > 25). The average door-to-CT time was recorded at 22 ± 6 minutes. Although this falls within internationally accepted benchmarks for trauma imaging, a statistically significant delay was noted in patients with higher ISS values (p = 0.002). This is clinically relevant, as prior literature emphasizes that door-to-CT times under 20 minutes are associated with improved survival rates, particularly in hemodynamically unstable or severely injured patients [10]. Contributing factors to imaging delays may include logistical inefficiencies, lack of prioritization protocols, and ED congestion. The implementation of a streamlined CT activation protocol, which allows direct transfer of trauma patients from ED triage to radiology upon activation, may reduce critical time intervals. Additionally, integrating CT scanners within or adjacent to the resuscitation bay-a model employed in high-performance trauma centres-could further expedite imaging and decision-making. The current average PRBC: FFP transfusion ratio of 1.5:1 does not align with evidence-based standards for damage control resuscitation, which advocate a 1:1:1 ratio of packed red blood cells, fresh frozen plasma, and platelets to mimic whole blood and mitigate trauma-induced coagulopathy [11, 12]. Imbalanced transfusion practices may compromise hemostasis, exacerbate bleeding, and increase mortality in patients experiencing massive hemorrhage. To address this, the adoption of point-of-care viscoelastic assays such as thromboelastography (TEG) or rotational thromboelastometry (ROTEM) is recommended. These tools provide real-time assessment of clot formation and stability, allowing for goal-directed transfusion strategies tailored to individual coagulation profiles [13]. Institutions equipped with such tools report not only improved transfusion accuracy but also reduced blood product wastage and complications. A significant gap in the current care model was the absence of a dedicated, multidisciplinary trauma team operating on a 24/7 basis. Variability in care processes and delays in decision-making likely stemmed from fragmented roles and inconsistent communication among care providers [14]. Numerous studies have demonstrated that formal trauma teams-comprising trauma surgeons, emergency physicians, anesthesiologists, radiologists and critical care nurses-lead to superior outcomes, including reduced ED length of stay, earlier operative interventions and decreased mortality [15, 16]. Implementing such a model would necessitate clear role delineation, standardized activation criteria and interdepartmental simulation training [17, 18]. The strengths of this study include its prospective observational design, which enhances temporal accuracy in capturing key resuscitative timepoints and interventions. Additionally, the use of standardized data forms ensured uniform data collection across shifts. However, notable limitations must be acknowledged. Being a single-center study, findings may not generalize to hospitals with differing patient loads, staffing models, or resources. Furthermore, long-term functional and rehabilitation outcomes were not assessed, leaving the impact of early interventions on patient recovery unquantified. Pre-hospital factors, such as time to first medical contact, field intubation, or inter-facility transport delays, were also not incorporated, potentially underestimating the burden of delays before ED arrival. The findings underscore several actionable priorities. First, the development of a protocol-driven, rapid CT activation system is essential to minimize time-to-diagnosis in severely injured patients. Second, the integration of viscoelastic testing within ED or trauma bay workflows can transform transfusion practices from reactive to precision-based. Third, hospital administration should prioritize the formalization of a 24/7 trauma team, underpinned by dedicated staffing, simulation-based training, and continuous performance audits. Lastly, establishing a quality improvement (QI) framework, including routine monitoring of key performance indicators (KPIs) such as imaging times, transfusion ratios, and ICU transfers, will allow institutions to iteratively refine trauma workflows. Regular audit-and-feedback cycles have been shown to foster accountability, enhance compliance with guidelines, and improve patient outcomes over time.

## Conclusion:

Management of polytrauma patients in India adheres to ATLS guidelines, but opportunities remain to reduce delays in imaging and optimize blood product utilization. Establishing dedicated trauma teams and protocol enhancements may translate into improved survival and resource utilization.
